# Tanzanian midwives’ clinical practices and experiences in caring for women with antepartum hemorrhage: a critical incident technique study

**DOI:** 10.1186/s12884-024-06802-7

**Published:** 2024-09-23

**Authors:** Anastazia Emil Dinho, Lena B. Mårtensson, Mattias Georgsson, Rose Laisser, Susanne Knutsson

**Affiliations:** 1 Archbishop Bishop Anthony Mayala School of Nursing, Department of Community and Maternal and Child health, Catholic University of Health and Allied Sciences, Mwanza, Tanzania; 2 School of Health and Welfare, Department of Nursing, School of Health and Welfare, Jönköping University, Jönköping, Sweden; 3 School of Health Sciences, University of Skövde, Skövde, Sweden; 4 School of Nursing, Midwifery and Social Work, University of Queensland, St Lucia, Brisbane, Australia; 5 Department of Health and Caring Sciences, Faculty of Health and Life Sciences, Linnaeus University, Växjö, Sweden

**Keywords:** Midwives, Midwifery, Critical incident technique, Clinical practices, Care of woman, Antepartum hemorrhage, Experiences, Caring

## Abstract

**Background:**

Antepartum hemorrhage (APH) is an obstetric emergency that complicates pregnancy worldwide and continues to lead to hemorrhagic conditions in parts of Tanzania. Midwifery education received by midwives consists theoretical knowledge on the subject but with no or minimal practical skills in the laboratory, which may reduce their practical capacity as graduated midwives. This study therefore aimed to explore midwives’ clinical actions and experiences regarding the care of women with APH in Mwanza region.

**Method:**

Qualitative, inductive approach with critical incident technique was used. Data were analysed using the critical incident technique, and a question guide consisting of eleven open-ended questions was used to collect data from 44 out of 60 midwives who graduated not less than one year. A total of 522 critical incidents, with 199 actions and 323 experiences, were identified and categorized into five main areas. Ethical approval was obtained.

**Results:**

Midwives’ clinical actions and experiences in caring for women with APH are affected by the knowledge and skills obtained during training at school. They have insufficient theoretical knowledge and practical skills, leading to inadequate identification of the problem and the implementation of care. A need for additional preventive care is described and structural issues, such as co-operation, referral to other instances, access to equipment and relevant treatments need to be improved.

**Conclusion:**

The actions taken to provide care for women with APH were related to their ability to identify problems, implement care and carry out structural initiatives. However, the midwives’ experience was influenced by an attempt to understand the seriousness of the situation and the existence of an organizational challenge. The results can provide knowledge and tools to improve midwives’ education and clinical practice and in the long run, prevent complications, improves health and minimize suffering in women with APH.

## Background

Ante partum haemorrhage (APH) is an obstetric emergency that endangers the lives of pregnant women and complicates both labor and birth. The risk factor for this complication include intrauterine foetal death, malpresentation, prematurity, low birth weight, congenital malformation, birth asphyxia, retained placenta, postpartum haemorrhage, anaemia and shock [1–3]. APH is defined as bleeding from or through the genital tract occurring at the beginning of the 24th week of pregnancy and prior to the birth of the baby [4]. It complicates 3–5% of all pregnancies and is among the leading cause of perinatal and maternal mortality worldwide [4]. Although maternal mortality declined globally, by approximately 38% from 2000 to 2017, 810 women still die daily due to complications resulting from pregnancy and childbirth [5]. Maternal mortality in Africa is a leading cause of death, especially in Sub-Saharan African regions, and accounted for approximately 70% of global maternal deaths in 2020; however, there was an average 3.0% decrease from 2000 to 2017 [6–8].

Tanzania is situated in the Sub-Saharan African region, and its maternal mortality rate in 2020 was 238 per 100,000 live births, but this figure has decreased by 9.16% since 2019 [9]. However, efforts need to be made in Tanzania to reach the Sustainable Development Goals target of 70 maternal deaths per 100,000 by 2030 [10]. Tanzania’s Health Management Information System reports from 2009 show that Mwanza was a leading region for maternal mortality in Tanzania and had (176) deaths, followed by Tabora (164) and Dar es Salaam (137) [10]. According to the Lake Zone maternal deaths report in 2022, among the seven regions of the Lake Zone, Mwanza was still the leading region for 75% of maternal deaths, while the remaining six regions carried only 25% of maternal deaths [11]. Furthermore, a 2019 report from the Mwanza Region Health Centers showed that 21.3% of women with complications related to pregnancy and birth had APH [12]. According to the register book of the Bugando Medical Centre (BMC), where obstetric emergencies were ranked from 2019 to 2022, APH was the 2nd most common obstetric emergency condition in 2019, 2020, 2021 and 2022, with 59 (23%), 62 (24%), 45 (26%) and 83 (21%) women, respectively, but still had a leading haemorrhagic condition. The leading obstetric emergencies were preeclampsia and eclampsia [13].

High-quality antenatal care for women depends on competent midwives to reduce the risk of APH. According to the International Confederation of Midwives, early detection and treatment or referral are essential competencies for midwives to provide high-quality antenatal care to maximize health during pregnancy [14]. The same competencies are also emphasized by the Tanzania Nurses and Midwife Council (TNMC) based on the World Health Organization (WHO) [15]. According to the TNMC, a graduated midwife should be able to identify deviations from normal pregnancy and initiate the referral process for conditions requiring higher levels of intervention to prevent complications [16]. Midwives also required to practice treatment process confidently, when providing preventive and promotive care for a normal pregnancy. Also, they need to have ability to detect complications, and carry out emergency measures, including treatment or initiation of the referral process for complicated cases [15, 17]. Midwives must interpret laboratory findings, analyze and synthesize data critically to identify gaps and areas for improvement, and, subsequently, plan and implement quality care [18]. Previous studies, such as Bhandari and Hunter [19], have shown that a poor outcome of care is among the leading factors associated with maternal mortality [20]. Other factors include poor management of women in 17% of the women treated in hospitals in Tanzania [2], a shortage of a skilled birth attendant [21], a lack of quality skills in midwives [22], and lack of evidence-based guidelines for the management of pregnant women [20]. However, morbidity and mortality rates can decrease and become more preventable. Mothers may have a great chance of survive if timely and adequate interventions are provided by a skilled birth attendant [6, 23–25].

Actions have been taken to empower pregnant women to lower their mortality rate. For instance, the Continuing Professional Development (CPD) program begins after basic training. The aim is to improve performance by continuously updating knowledge, skills and attitudes, and among the strategies used to ensure quality care is the involvement of midwives in CPD. With the continuous use of CPD assisted by their employer, midwives retain their capacity to practice safely, effectively, competently and legally within their scope of practice [26]. The government of Tanzania and other partners, such as the Johns Hopkins Program for International Education in Gynecology and Obstetrics (JHPIEGO), also, provided technical assistance by developing the Essential Maternal and Neonatal Health Guidelines in 2016. They have also conducted training and supervision using different midwifery simulations and reduced maternal morbidity and mortality rates [26]. However, among the leading obstetric emergencies facilitated during the training, APH was solely conducted theoretically; it is not known whether the midwives could obtain the required practical competencies to care for women with APH. Generally, midwives were expected to have essential competencies in emergency care after completing the course.

These factors are associated with maternal mortality and indicate that pregnant women are still at risk of not receiving necessary care when experiencing APH, leading to the health and wellbeing of these women being at risk [2, 20–22]. There is a need for midwives to continuously practice, simulate, prepare, and use the standards during the provision of care to women with APH to prevent complications. Utilization of nursing process for the provision of care to the woman with APH is emphasized, under the following clinical actions; assessment of the women with APH, planning for care, implementation and evaluation. Therefore, this study aimed to explore midwives’ clinical actions and experiences regarding the care of women with APH. The results may provide knowledge and tools to improve midwives’ education and clinical practice. In the long run, complications may be prevented, suffering may be minimized, and health and well-being may increase.

## Methods

### Design

This study used a qualitative, inductive approach based on the critical incident technique (CIT) [27, 28]. A critical incident is understood as a retrospective situation or event participated in or observed by a participant, which should have a beginning, an end and a significant outcome. The results can either be positive or negative [29]. CIT was used to visualize both the experiences and actions taken [28, 29] regarding the care of a pregnant woman with APH. Application of CIT enhanced rich contextual information from the midwives, which obtained to get critical requirements for care the woman with APH. The method allowed researchers to understand the complexities of midwives’ actions and experiences on APH care, as well as interactions between midwives and other medical personnel [27]. Furthermore, the participants were encouraged to remember a critical incident and describe the scenario broadly.

### Setting

This study was conducted in the Mwanza region, one of 30 regions of Tanzania. According to the Tanzania National Bureau of Statistics census of 2022, the population is 3,699,872 out of the country’s population of 65,497,748 [30]. Children younger than one year are 3.4% of the total population of the Mwanza region [31]. The standard health center comprises an outpatient and inpatient department with 24 beds for males and females. There are also obstetric theatre and diagnostic services. The health center serves a total of 50,000 people. The minimum staffing level is supposed to be one medical doctor, one assistant medical officer, one radiographer, two laboratory technicians, ten midwives and six medical attendants [32]. The number of staff reported from the ten health centers in Mwanza region shows that these centers were understaffed during the data collection.

### Participants

This study included midwives from ten health centers in the Mwanza region. The criteria for selecting ten out of 28 health centers in the area was because they are large quantity sites for APH and had an average of more than 83 deliveries per month. The selection was made in order to increase the likelihood of receiving midwives’ stories about the care of women with APH. The inclusion criteria in the study were midwives who had completed midwifery diploma school and who had cared for women with APH. The regional medical officer received information about the study, signed the approval for the data collection and informed the five district executive directors (DEDs) to allow the process to be performed in their districts. The DED then sent the information letter about the study and the approval for data collection to the respective health centers through the district medical officers (DMO). The DMO handed the matter to the medical doctor in charge in each selected health center, and after that, the medical doctor in charge informed the midwives of the health center. Purposive sampling was performed to reach midwives who had experienced a critical incident during their working process. Therefore, the hospital matron assisted in the identification of midwives who met the inclusion criteria [33]. The hospital matron provided verbal and written information about the study to 60 selected midwives, who were asked if they wanted to participate. Forty-four midwives agreed to participate, and the hospital matron provided the contact information of these midwives to the first author. The first author contacted them by phone to inform them about the study and its aim and coordinate their participation according to the duty roster.

The matron allowed the participants to meet individually with the researchers during working hours at their working places. The participants signed a written informed consent form regarding their participation before the data collection, which took place in a side room near the maternity ward or at an office depending on the hospitals’ infrastructure.

The required sample size for CIT is based on the volume of information needed. According to Schluter et al., the number of incidents is recommended to be no less than 50. In contrast, while Flanagan recommends that 50 to 100 incidents be collected depending on the complexity of the research question [27, 34]. A collection of 50 or more incidents leads to differences in participants’ expression and ensures that sufficient and quality data are gathered [27]. After collecting reports from 44 participants’, the researchers considered that a sufficient number of critical incidents had been obtained which was enough to achieve the goal of more than 100 critical incidents. The demographic characteristics of the participants are shown in Table 1.

**Table 1:** Characteristics of participants

Age	Participants	Sex		Work experience (years)			Where did you learn about care of woman with APH?					
		Male	Female	< 1	1–5	> 5	MS	SL	M	F	OJ	S
18–25	2	2	0	1	1	0	2	0	1	0	0	0
26–35	26	13	13	1	11	14	23	2	13	4	1	2
36–40	14	2	14	0	1	15	16	0	9	3	1	0
Total	**44**	**17**	**27**	**2**	**13**	**29**	**41**	**2**	**23**	**7**	**2**	**2**

APH = Ante Partum Haemorrhage, MS = Midwifery School, SL = Skills Laboratory, M = Midwives, F = Friends, OJ = On job training, S = Seminar

### Data collection

**Fig. 1:**
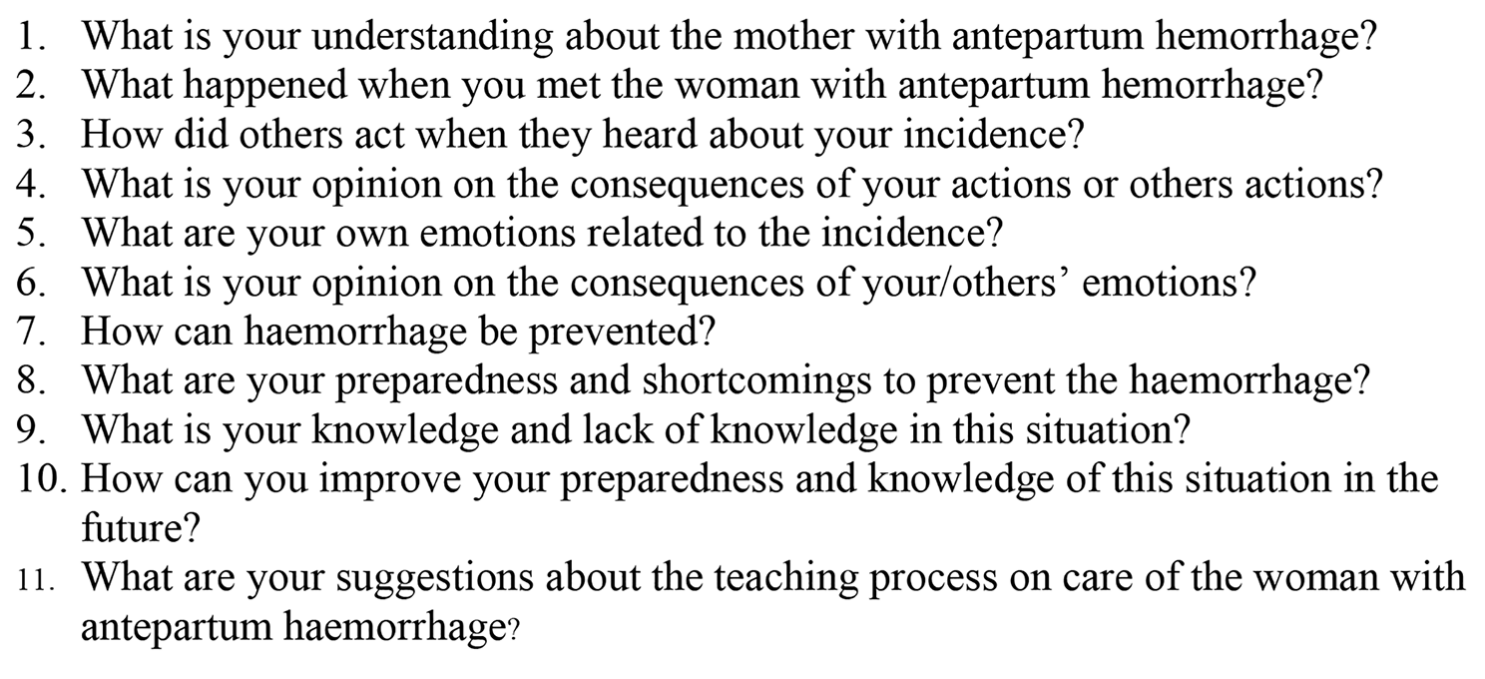
Question guide

The data were collected using a question guide comprising eleven open-ended questions focusing on midwives’ care of women with APH. The CIT was composed of the CIT structure and was given to the participants (Fig. 1).

These questions align with Fridlund et al. and Schluter et al. [27, 29]. The question guide was piloted with five midwives working at the closest health center; these midwives were not included in the main study. The pilot study yielded minor clarifications and sentences adjustments to enhance the questions clarity [35]. The participants were instructed to respond in writing (self-reporting) to the incident providing a detailed description. The focus was on critical events that had either a positive or negative impact on them when they provided care to the woman with APH during admission, identification of the problem and overall emergency care [28, 33]. The participants completed the question guide in approximately 30 min and then handled it to the first author in-person. The data were collected from August to November 2021.

### Data analysis

The data analysis process was influenced by Flanagan, Fridlund et al. and Holmqvist et al. [28, 29, 35]. The authors started the process of analysis by defining the terms “action” and “experience” [35]. In this study, action meant care which provided to the woman with APH, but the experience was what the midwife practiced as meaningful about the care of the woman with APH, or the impression left to midwives after practical contact or observation gained from a care situation involving a pregnant woman with APH.

All authors read the written text from the midwives several times to become familiar with the content. Actions and experiences were analyzed separately. Each identified action was condensed and coded based on content. Codes with similar content were grouped to form subcategories, and then, by abstraction, the subcategories were grouped into categories. After that, the categories were grouped into main areas. The categorization and grouping of the samples into main areas were based on the content where similar content was grouped together. The same procedure was used for the experiments [28, 29, 35]. All authors were involved in all steps taken in the analysis, and the entire research group discussed the text until a consensus was reached.

### Ethical considerations

Ethical approval from CREC/468/2020 was obtained and approved by the Joint Ethical Committee of the Catholic University of Health and Allied Sciences (CUHAS) and Bugando Medical Center (BMC), and the study adhered to the Declaration of Helsinki ethical principles for medical research [36]. Permission was also provided by the Regional Administrative Secretary for data collection in the region and the District Medical Officers for the five district hospitals for data collection in their health centers. Considering ethical research principles [36, 37], written informed consent was obtained from all participants prior to data collection. They were informed about the aim of the study, confidentiality and the possibility of withdrawing at any time.

## Results

In this study, 60 midwives who had completed a diploma in nursing and midwifery and who were experienced in caring for women with APH at ten health centers in the Mwanza region were asked to participate. A total of 44 midwives participated. A total of 522 critical incidents, divided into 199 actions and 323 experiences, were identified. Three main areas of midwifery actions positively or negatively influencing the care of women with APH were identified: *identification of the problem, implementation of care* and *structural initiatives* (Table 2). Experiences from the midwives formed two main areas: *striving for an understanding of the seriousness of the situation* and *striving for an organizational challenge* (Table 3).

### Actions

The midwives took different actions regarding the care of the woman with APH, but these actions were often incomplete and irregular and involved only a few midwives. The actions comprised of three main areas, six categories and 14 subcategories as shown in Table 2.

**Table 2:** Summary of midwives actions of care for the woman with antepartum hemorrhage

Critical incidents (quotes)	Subcategory	Category	Main area
Received with APH//Lethargic appearance//Pale +++, Hb 4 g/d//The blood pressure upon arrival was 60/40 mmHg with Pulse rate (PR) 112 6pm (40)	Performing general observations (16 + 28 = 44)	Assessment to identify obstetric problems (67)	Identification of the problem (88)
Test for blood group and get blood early//The results were O + ve, Hb 10 g/dl (35).	Carryout investigation (12 + 11 = 23)		
Gestation age was seven months and Fundal Height (FH) 29/40 (21)	Measuring fundal height and gestational age (02 + 07 = 09)	Assessment to identify Fetal condition (21)	
The Fetal Heart Rate (FHR) was absent (21)	Performing observations of fetal heart rate (04 + 09 = 12)		
We set the drip//given a blood transfusion but still pale (16)	Uplifting blood volume (10 + 12 = 22)	Giving treatments (64)	Implementation of care (73)
Catheter to measure urine output (35)	Inserting the catheter (05 + 07 = 12)		
Prepared for surgery// The women operated (01)	Performing operative care (10 + 13 = 23)		
Close monitoring (21)	Performing post-operative care (03 + 04 = 07)		
The doctor started medication and was given a bed rest (21)	Ordering rest (02 + 03 = 05)	Preventing further complications (09)	
I did not perform PV examination (08)	Excluding PV examination (02 + 02 = 04)		
I shout for help and we help each other during the procedure to prevent the maternal death// Teamwork in emergence situation to prevent death (38)	Consulting colleagues (09 + 14 = 23)	Co-operating with other persons (29)	Structural Initiatives (38)
I called for help from a doctor//I called for help from lab person (35)	Consulting other professionals (02 + 04 = 06)		
Early referral to higher center//I gave her a drip and referred her (44)	Discharge to other healthcare institutions (04 + 04 = 08)	Referral to other instances (09)	
Discharged on Haemovit syrup (24).	Discharge to home (0 + 01 = 01)		

APH – ante partum haemorrhage, Hb – hemoglobin, FH – Fundal height, FHR – Fetal heart rate, PV – Per Vaginal, Numbers stands for (male + female = Sum)

#### Identification of the problem

*Assessment to identify obstetric problems* concerns the kind of general observation that midwives perform. The vital signs, bleeding, pallor, abdominal pain, state of consciousness and general weakness of the woman were assessed. There was an irregularity in assessing vital parameters by the midwives, and often incomplete vital signs were assessed, such as assessing only one or two observations. The midwives also reported that the woman had slight to severe bleeding, abdominal pain and general body weakness, including a state of consciousness. The descriptions from the midwives showed that assessments and checks were used inconsequently and sometimes not at all. A laboratory test was occasionally performed to confirm the diagnosis. The midwives sent the woman for haemoglobin (Hb) and other tests for blood transfusion, but the women were rarely prescribed an ultrasound examination to determine the cause of bleeding. The descriptions from the midwives showed that laboratory tests and ultrasound were inadequate.

*Assessment to identify fetal conditions* involved the midwife’s examination of the fetus to determine its size and age. The midwives described performing this examination; however, midwives’ description showed that this was done irregularly and incompletely. The fetal heart rate was rarely checked, and abnormalities in fetal heart rate were observed in those with blood loss. The descriptions from the midwives show that assessments and checks of the fetus were irregularly performed and seemed to be completely forgotten by some midwives.

#### Implementation of care

*Giving treatments* Treatment refers to routine work in providing care to pregnant women to relieve them from dangerous situations. The midwives regularly lifted the blood volume via intravenous infusion and blood transfusion. In cases of emergency, if the medical doctor was late, the nurses administered an intravenous infusion to save the life of the mother and baby. They also assisted in inserting a urinary catheter to relieve discomfort, which is a part of perioperative care. To prevent additional complications, they frequently made decisions regarding the performance of operative care in this emergency. The midwives irregularly performed continuity of care, which included close observation of the woman and baby to prevent danger, from erupting. The description from the midwives showed that primary emergency care was partly provided and that continuity of care was inconsistently provided.

*Preventing further complications* involves the actions taken by midwives to prevent dangerous situations that may occur during pregnancy. The midwives encouraged the woman to rest to stop bleeding. They also excluded vaginal examination because this procedure is dangerous. After all, it can cause severe hemorrhage and worsening of the condition. These preventing actions were performed irregularly and were rarely mentioned by the midwives.

#### Structural initiatives

*Cooperation with other persons* describes midwives’ actions to seek help from other midwives and medical attendants during care provision, especially in emergency care, such as severe bleeding. The midwives frequently consulted colleagues to work as a team to save the woman´s and the baby´s life. They said they asked for help and that the help came very quickly. The midwives also consulted other professionals, who described that they called for help from the medical doctors and the lab. The midwives also described working as a team with other co-workers as necessary for saving the life of the woman and fetus.

*Referral to other instances* describes the midwives’ proactive actions to refer the woman to other instances. The midwives participated in decisions about referral to other healthcare institutions for more specialized care since the care at the current institution could not manage the situation. The midwives also participated in decisions about sending the woman back to her home after recovery.

### Experiences

The midwives’ experiences of caring for the woman with APH resulted in either a bad or good outcome. However, only a few midwives had good outcomes. The experiences comprised two main areas, five categories and 17 subcategories (Table 3).

**Table 3:** Summary of midwives experience of care of the woman with antepartum hemorrhage

Critical incidents (quotes)	Subcategory	Category	Main area
Is a danger sign for the mother and the unborn child (32)	Understood one component of definition (6 + 16 = 22)	Uncertainty in the diagnosis (43)	A strive for an understanding of seriousness of the situation (243)
It is an act of vaginal bleeding from a pregnant woman before giving birth (31)	Understood two components of definition (6 + 4 = 10)		
Is a bleeding from or into the genital tract occurring from 24 weeks of pregnancy and prior to the birth of baby. The most important causes of APH are placenta previa and placenta abruption. (40)	Understood three or more components of definition (5 + 6 = 11)		
Adequate education for mothers during pregnancy //Parents should be given adequate education about danger signs (30)	A need of knowledge in women (15 + 27 = 42)	A crucial importance of knowledge (138)	
Education should be provided in the community, antenatal clinic so that mothers can be at the center before they reach this critical stage of loss of consciousness (32).	A need of knowledge in community (02 + 09 = 11)		
They surprised gave birth prematurely is uncommon situation // It is important to have proper knowledge on bleeding during pregnancy (06).	A need of knowledge in midwives (04 + 10 = 14)		
I was just completed my course, I wondered what I should do as a PPH? No, the child was still in // Failure to make service decisions //To understand what we are doing // Attend regular self-development training // Learn more APH (11).	A need of continuing education (12 + 19 = 31)		
It is advisable to have practical exercises regarding bleeding during pregnancy // More practical training // Use practical methods as it is in PPH (06).	A need of quality education to student midwives (17 + 23 = 40)		
The blood stopped and she later gave birth safely // they all recovered // They commended for the good service, because mother and baby were safe (25).	Joyful feelings (12 + 17 = 19)	An emotional touch (62)	
The amount of blood that was coming out // Dead on the way to hospital // They were deeply saddened by the death of the mother and child on the way to hospital // Preventable maternal death (27)	Depressing feelings (16 + 27 = 43)		
Teamwork to be available (36).	A cry out for help (04 + 10 = 14)	A need of teamwork (17)	An organizational challenge (82)
I was alone (26)	An incomplete team (01 + 02 = 03)		
They had no money to rent a car, they struggled to raise money and rent the motorcycle, The facility should have own transport to transfer the patients when needed (26).	Inadequate transportation (0 + 04 = 04)	A structural issue (65)	
It is better to have an emergency tray // Limitations are the lack of some emergency equipment // to Have an emergency tray nearby (18).	Inadequate equipment (10 + 18 = 28)		
Increase providers, medicines and blood // We had no blood reserves at the centre // having blood stocks and other supplies (26)	Inadequate treatment (05 + 08 = 13)		
Shortage of staff // Manpower should be increased in hospitals / clinics // Manpower including adequate providers (04).	Limited number of staff (03 + 04 = 07)		
Nurses make quick and accurate decisions // Nurses to be careful in this dangerous disease which can lead to death (11).	A need for a more structured preventive care (07 + 06 = 13)		

APH – ante – partum haemorrhage, PPH – post – partum haemorrhage, Numbers stands for (male + female = Sum)

#### A strive to understand the seriousness of the situation

*The midwives described uncertainty in the diagnosis* of APH regarding the identification of different signs, such as bleeding, pallor, abdominal pain and dizziness. The causes and occurrence of APH are well known. However, midwives have difficulties in explaining the components of the definition of APH, which should include “the bleeding from the uterus through the vagina that occurs in the second half of pregnancy (after the 24th week) and before the birth of the baby; it is a life-threatening situation and one of the obstetric emergencies that complicates labor and delivery”. For example, some midwives explained that, this bleeding was “caused by placenta”. The midwives do not see the situation as serious since they fail to identify vital signs and causes of bleeding or high blood pressure. The description from the midwives shows that they are endeavoring to help but a confident ability to diagnose APH is missing. Their uncertainty undermines their understanding of the situation´s seriousness.

The midwives’ experiences *A crucial importance of knowledge* concerning providing education to women, the community and themselves on APH and its prevention. The midwives described that it is essential to provide knowledge to women, especially on APH complication prevention and self-care. The midwives also described the importance of health education to the community on preventive measures for APH complications. Their description showed they experienced inadequate knowledge among the women and the community and proposed the health education. Moreover, they described that their fellow midwives needed more knowledge about APH and that they had insufficient experience with APH care. Therefore, they suggest continuing education even after school. They also described that it would be exemplary if the nursing schools could provide theoretical and practical education on APH to midwifery student. The midwives experienced midwifery students being partially taught, resulting in difficulties in practicing after school was completed.

The midwives *described receiving emotional touch* while providing care to the women with APH. They described those feelings, such as happiness and sadness that occur during the care process. They feel happy when women deliver safely, and they sometimes feel sad due to the death of the woman and baby. Midwives frequently experienced feelings of depression after receiving adverse outcomes during and after the provision of APH care.

#### An organizational challenge

The midwives described feeling *a need for teamwork.* They express that functioning in the utilization of joint efforts in emergencies is vital and stresses the need for such efforts. The midwives described situations in which they had to cry out for help and wait for help when they found themselves unable to perform several tasks alone to save the life of the woman. The midwives’ descriptions showed that they were stressed when help was delayed and described the importance of efficient teamwork when managing APH.

The midwives’ experience shows that their care for women with APH is affected by *a structural issue.* This issue concerns human and nonhuman resources that support the provision of quality care to pregnant women; these resources are inadequate and can affect the outcome of care. For example, the midwives may experience transportation problems in their working area, may irregularly have difficulties transferring women to health centers for specialized care.

The midwives also complain about inadequate equipment. They described that they commonly miss critical equipment for the procedure due to a lack of equipment or difficulties finding or accessing to them.

They further argued for the inadequacy of treatment. The midwives described that some medicines, blood and other supplies were limited in dispensaries and health care centers.

They also experienced an inadequate number of staff. The midwives described that the limited number of staff members may result in incomplete teamwork, which they thought was essential in emergency care.

Midwives describe the need for more structured preventive care. They experienced problems with slow decision-making and carelessness in midwifery care, which goes against preventive care for women with APH. Therefore, they suggested being quick and accurate in decision-making and being careful when providing care.

## Discussion

Using the critical incident technique this study highlighted midwives’ clinical actions and experiences in caring for women with APHs. The knowledge and skills related to APH, preventive care, structural challenges and teamwork to support care are essential areas to be addressed.

### There is a need for more knowledge and skills

The findings show that midwives had inadequate theoretical knowledge and practical skills regarding APH. However, midwives have trouble in identifying the problem, by definition, causes and occurrence, which leads to difficulties in providing the required care. The midwives in Malawi describe the same: they received important information about APH, but there were deficiencies in some critical areas, such as the definition of APH and predisposing factors [38]. The midwives in the present study had difficulties in diagnosing APH, and the findings revealed that they also had inadequate assessment skills. The assessments and checks were performed inconsequently and were sometimes not performed at all, which led to a delay in diagnosis. A previous study by Shimoda blames the majority of midwives for processing assessments and judgments in brief and without reflection [39], and a study about midwives in Malawi showed that midwives have deficiencies in assessing women [38]. The findings in the present study also showed that the participants rarely mentioned excluding vaginal examination, which is an essential preventive action for severe bleeding. To prevent further bleeding, the Ministry of Health and Social Welfare (MoHSW) emphasizes the avoidance of digital vaginal examination to women with APH [21]. The midwives in the present study occasionally conducted laboratory tests and ultrasounds. These investigations should be performed after the woman arrives at the facility. Mukherjee emphasized that ultrasound scanning is an effective screening tool for detecting placenta previa in mid-pregnancy; if not taken in time, the diagnosis will be delayed [40], and care will be delayed; subsequently, the women’s condition could deteriorate.

Most of the midwives exhibited incompetency in providing basic emergency care and continuity of care. This finding aligns with the study performed in northern Tanzania in 2009, where the staff lacked quality care provision for emergency obstetric care [41]. The problem of incompetency in provision of emergency care is in line with the study done in Ethiopia [42], If this continues, it can endanger the life of the woman. In 2021, the WHO developed ten milestones to prevent deaths, among which *quality of care* was mentioned, emphasizing effective maternal care [5]. Therefore, deaths can be prevented by having a competent and skilled midwife jointly with other human and nonhuman resources [22].

The results also show that midwives refer women to other instances but admit they must be quick and make accurate decisions. The same happened in Burkina Faso, where there was a delay in decision-making and the referral process contributed to increased maternal morbidity and mortality [1]. According to a study about perinatal mortality due to APH it seems to be above 50% in developing countries including Tanzania [43]. Deficiencies in the referral of women have also been observed in other places [38].

Midwives need to train midwifery skills to be able to manage normal deliveries, diagnosing, managing and/or referring women with obstetric complications [22]. The midwives in the present study explained that the learning process on APH begins in midwifery schools and that, during training, it is well-supported by midwives in clinical areas. The midwives in Dublin experienced the same, they received support from midwives during their practice in the clinic [44] to increase their competence. The results of the present study revealed that midwifery students receive more theoretical information about APH education and have minimal practice in the skills laboratory, which is expected to reduce their practical capacity in relation to inadequate skills that could endanger the health of the woman and fetus. The same was discussed by Sorensen, who suggested that a lack of quality skills for midwives is among the contributing factors to maternal deaths [22]. In Tanzania, teaching personnel use the facilitators’ guide, which is uniform and applied in all midwifery schools. The guide for APH lesson plans consists of two teaching methods: lecture-discussion and case study. However, the time allotment for practice is not indicated [45, 46], so the teaching personnel can decide how to teach practice on APH. The findings show that the challenges in clinical practice are associated with problems in selecting methods for teaching and learning. Other studies emphasize that various teaching approaches develops learners’ competence [47, 48]. Since the facilitators’ guide misses, some vital information for competence building, it is not known whether midwifery educators are modifying the existing APH lesson plan to build competence. This finding aligns with the findings of studies performed in the Democratic Republic of the Congo, where most of midwifery tutors and preceptors had deficiencies in midwifery competence, which resulted in barriers to delivering quality midwifery education [49]. This is also in line with the results of the study done in Ethiopia where insufficient pre-service and in-service training on care of emergency were found [42]. Since the findings highlighted the insufficiency of APH knowledge and skills after completing their courses, the midwives suggested that midwifery schools to provide quality education where theory should be taught together with practice and additional time to be given for practical education to gain competence. Midwifery students and experienced midwives in Sweden also show the same: the current content in midwifery education related to clinical competence needs an extension of time, especially in emergencies such as the APH [50]; this would build more competence for students because educational and clinical interventions are among the strategies to prevent deaths due to obstetric haemorrhage [51]. In this case, the midwifery curricular needs revision with special consideration of the steps of APH care to enhance quality care for women with APH. Long and Kolb’s theory of experiential learning advises focusing on real-world experiences during the learning process and emphasizing learning through application [52]. Therefore, simulation-based learning must be introduced as a method for teaching APHs in schools and clinics. Simulation-based learning will be organized such that the student can practice only how to handle emergency care during admission, and how to deal with priorities in this situation. The focusing on theoretical teaching alone may have contributed to the midwives’ difficulties in identifying APH. Special programs need to be prepared according to Kolb’s theory, following the established standards and the use of emerging evidence [5, 52]. The midwives in the present study admitted a gap in knowledge and skills after completing their course and suggested frequent on job training, as per the TNMC guidelines [53], to improve their skills. The same was recommended for Malawian by Kapyepye to initiate an in-service education programme for midwives to increase their knowledge of complications and danger signs during pregnancy [35]. The findings also showed that developing systems that revised the learning experience to prevent the reoccurrence of the same negative experiences could help to improve provision of care, which is also emphasized by the WHO [5]. Improving knowledge and skills for midwifery students depends on the competence of midwifery educators and midwives.

According to the findings, care provided by midwives does not follow the established standards, and there is no step-by-step guideline to follow for APH care in midwifery schools. In 2016, Nyamtema explained that Tanzania lacked evidence-based guidelines for enhancing maternal and perinatal health in remote areas, leading to poor performance and poor outcomes [20]. Similarly, in 2017, at Muhimbili Consultancy Hospital, the substandard in emergency care was among the causes of maternal mortality [2]. Kapyepye explained that, after identifying that the care of women with hemorrhage during pregnancy was associated with poor outcomes in Malawi, they ultimately developed guidelines for the provision of quality care [38]. Such APH quality guidelines are needed worldwide.

### There is a need for additional preventive care

Our study reported on the occurrence of maternal deaths before care. The results also showed that women and other community members have inadequate knowledge of APH in identifying danger signs and birth preparedness. The findings revealed the women’s tendency to come late to the hospital and the death of women which sometimes occur on the way to the health facility. This finding is in line with the study performed by Bhandari, in which late decision-making for households in developing countries affected the management of women in emergencies [19]. The midwives suggested providing health education to pregnant women and other community members. In Kenya antenatal and general community education emphasized that, if the women experiences vaginal bleeding during pregnancy she must urgently seek health care [51]. Previous studies have shown that if community members are aware of danger signs, timely and adequate intervention can be provided to women in hospitals, and death can be prevented [23, 54]. Health facilities and communities should be aware and take relevant action to address three delays in providing care to women: delays within the family and community in the decision to seek care, delays in getting into transport, and delays within health facilities [22]. In relation to transportation delays, health care workers in Kenya were advised to educate pregnant women about obstetric haemorrhage and promote transport plans [51] Moreover, the community should be aware of the importance of hospital attendance and positively perceive health facilities [19]. An in-depth review of APH-related deaths is needed to determine appropriate approaches to ensuring the safety of pregnant women [23].

Therefore, providing health education to pregnant women and other community members is essential when they attend hospitals/clinics. Moreover, in school health programs and political meetings, early identification of the emergency is essential for pregnant women to prevent complications.

### There is a need to raise awareness of the challenges associated with nonhuman resources

The results showed that midwives experience challenges regarding structural issues (nonhuman resources), such as inadequate equipment for procedures, inadequate treatment and unreliable transport to transfer women to health facilities for specialized care when providing care to women at health centers. A study in Kenya [51] advised health facilities to have an emergency transport to be onsite for transfers. Olsen described that, among the barriers to implementing emergency care in Tanzania was a shortage of vehicles in health facilities [41]. which also was an issue described in Ethiopia especially in the rural area and also in other low income countries [42, 55], and generally inadequate transport facilities are also responsible for the utilization of emergency obstetric care in developing countries [19]. In Tanzania, families must have an individual birth plan, complication preparedness, and transport preparation during pregnancy [21], and to avoid difficulties the woman needs to be referred to a proper health facility in time. Another study [1] shows that women were referred to a specific hospital, which was inappropriate because the hospital is poorly equipped with materials and has a limited drug supply. A study made in Kenya also describe a need to improve supply of drugs, consumables and equipment for obstetric care for a more positive result of women’s health [51]. Olsen [41], also explained that a shortage of basic supplies affects the provision of care in emergencies. In Nigeria, it is described, that the lack of safe blood transfusion services contributes to maternal mortality [56] the visited health centres in this current study´s result described the same problem.

A well-equipped facility with a scan will facilitate quick management [57], but poorly equipped health facilities are common in developing countries [19, 20]. The equal distribution, access, appropriate use, and maintenance of equipment need to be emphasized in health facilities to facilitate high-quality care [5]. There is no quality performance without equipment or supplies. The institutional management must work on this [5]. Health facilities have to raise awareness about this and utilize the available resources properly to improve, if possible, the accomplishment of the required tasks. Additionally, the government should be encouraged to consider structural issues in its plans to prevent unnecessary deaths.

### There is a need for additional teamwork

The results of the present study revealed the importance of good collaboration among available health professionals; close cooperation facilitates smooth emergency care. Bhandari emphasized that teamwork motivates staff and improves and maintains quality care. A shortage of human resources affects the provision of quality services [19]. However, the current study revealed that, midwives sometimes experience loneliness and become stressed when help is delayed due to the absence of team members such as doctors and laboratory technicians, who are expected to support care during emergencies. The Ministry of Health in Tanzania described the same situation of staff shortage, which occurs in some health facilities in the country [21]. In Karachi, Pakistan, midwives perceived an increased workload due to a shortage of staff [58]. However, in 2021, the established sixth milestone by WHO suggested that, the countries to increase the number and retention of skilled health personnel for maternal health to prevent maternal mortality [5]. Inadequate human resources affect teamwork, especially when managing women in emergencies. Results of this study also showed that teamwork needs to be improved. The different professions should cooperate more and work as a team. The MoHSW required midwives to shout for help from other staff to jointly help each other to save the life of the woman with APH [21] to maintain teamwork. Human resources are an essential aspect of providing quality care, and the government should be encouraged to consider this issue to employ a sufficient number of trained staff members to prevent unnecessary deaths.

### Limitations

The limitations identified in this study included the fact that, the study depended on an action or experience remembered by the midwives. Since Critical Incident Technique (CIT) relies on individual memory, honest and accurate information is required during reporting; otherwise, the incidents reported by midwives may be inaccurate or unreported [29, 35]. Some midwives were not interested in participating, so it was challenging to convince them. In some cases, APH is considered a rare event, making it difficult to report. In this case, more common events that they faced could have been missed. The study process also took time because the analysis process was very demanding and required several steps, which is a common drawback of the CIT method [28].

The transferability of the results to other geographical areas can be seen as possible because there are several health centers in Tanzania where midwives are taught using the same curriculum and using almost the same teaching materials. Therefore, the applicability of this study to other contexts are also possible [33].

## Conclusion

Midwives’ clinical actions and experiences in caring for women with APH are affected by inadequate theoretical knowledge and practical skills obtained during training. Therefore, they need more knowledge and skills in caring for women with APH. Special programs need to be identified to develop both pre-service and in-service training, with emphasis on simulation-based learning in realistic case-scenarios included in order to improve skills and confidence.

The midwives also found preventing the occurrence of APH complications, such as maternal deaths, necessary because of the reported deaths of some women before initiation of treatment. Furthermore, they needed to be aware of resource challenges and structural issues preventing them from providing women with quality care. Close cooperation with other professionals is essential for enhancing teamwork and facilitating the smooth execution of emergency APH care for women. If all these steps are implemented, quality care will be provided to the women with APH to prevent complications and improve their health.

## Data Availability

The data are available from the authors upon reasonable request.

## Abbreviations

APH: Ante Partum Haemorrhage
TNMC: Tanzania Nurses and Midwife council
WHO: World Health Organization
CPD: Continuing Professional Development
CIT: Critical Incident Technique
DED: District Executive Directors
DMO: District Medical Officers
CUHAS: Catholic University of Health and Allied Sciences
Hb: haemoglobin
FH: Fundal height
FHR: Fetal heart rate
PV: Per Vaginal
PPH: Post-Partum Haemorrhage
MoHSW: Ministry of Health and Social Welfare
